# Willingness to disclose sexual orientation and gender identity on federal government surveys: A community-based research study with gay, bisexual, transgender, and queer men and nonbinary and Two-Spirit people in Canada

**DOI:** 10.17269/s41997-025-01127-0

**Published:** 2025-10-17

**Authors:** Kai Jacobsen, Coady Babin, Aki Gormezano, Everett Blackwell, Benjamin Klassen, Rob Higgins, Kiffer Card, Olivier Ferlatte, Nathan J. Lachowsky

**Affiliations:** 1 Interdisciplinary Studies Graduate Program, University of British Columbia, Vancouver, BC Canada; 2 School of Public Health and Social Policy, University of Victoria, Victoria, BC Canada; 3 Community-Based Research Centre, Vancouver, BC Canada; 4 Faculty of Health Sciences, Simon Fraser University, Burnaby, BC Canada; 5 École de Santé Publique de L’Université de Montréal, Montréal, Canada; 6 Centre de Recherche en Santé Publique, Université de Montréal Et CIUSSS du Centre-Sud-de-L’Île-de-Montréal, Montréal, Canada; 7 Faculty of Human and Health Sciences, University of Northern British Columbia, Prince George, BC Canada

**Keywords:** Sexual and gender minorities, Sexuality, Bias, Surveys and questionnaires, Canada, Minorités sexuelles et de genre, Sexualité, Biais, Enquêtes et questionnaires, Canada

## Abstract

**Objectives:**

Government-conducted population health surveys are important sources of data on health inequities for gay, bisexual, transgender, and queer men and nonbinary and Two-Spirit people (2S/GBTQ+). There is limited understanding of how vulnerable these surveys are to misclassification bias resulting from participants’ reluctance to disclose their sexual orientation and gender identity. 2S/GBTQ+ people may be more willing to participate in community-based surveys, where they might feel safer disclosing their minority sexual orientation or gender identity than they would on a government survey. We sought to understand whether the proportion of 2S/GBTQ+ people who would disclose their sexual orientation on a government survey changed between 2012 and 2019 survey cycles, as well as the proportion of trans, nonbinary, and Two-Spirit participants who would reveal their gender identity, and the demographic factors associated with both.

**Methods:**

We analysed data from the 2012 and 2019 cycles of Sex Now, a repeated cross-sectional Canada-wide online survey on the health and well-being of 2S/GBTQ+ people conducted by the Community-Based Research Centre. We computed frequencies and prevalence ratios of the likelihood of disclosing sexual orientation and gender identity on a Statistics Canada survey by a variety of demographic variables.

**Results:**

We found that in 2019, 86.0% (95% CI [85.4, 86.7]) of all participants would reveal their sexual orientation, a significant increase from 2012 (69.5%, 95% CI [68.5, 70.4], Δ = 16.6%, 95% CI [15.4, 17.8]). However, participants who identified as bisexual, straight, or heteroflexible; who were in a relationship with a woman; or who were not “out” were less willing to reveal their sexual orientation. We found that 85% of trans men, nonbinary, and Two-Spirit participants would reveal their gender identity, which was more likely among those living with HIV or aged 19–29 years old.

**Conclusion:**

These findings suggest that government datasets may significantly misclassify and underestimate the population size of 2S/GBTQ+ individuals. Persistent mistrust of government institutions within this community may exacerbate underreporting and non-disclosure, underscoring the need for research into methodologies that can enhance trust and improve the accuracy of population estimates. Researchers using existing government datasets should consider using statistical methods to account for potential misclassification error.

**Supplementary Information:**

The online version contains supplementary material available at 10.17269/s41997-025-01127-0.

## Résumé

**Objectifs:**

Les enquêtes gouvernementales sur la santé des populations sont d’importantes sources de données sur les iniquités en santé chez les hommes gais, bisexuels, transgenre et queer et les personnes non binaires et bispirituelles (2S/GBTQ+). Or, on en sait peu sur la mesure dans laquelle ces enquêtes sont vulnérables au biais d’erreur de classification résultant de la réticence des personnes participantes à dévoiler leur orientation sexuelle et leur identité de genre. Les personnes 2S/GBTQ+ peuvent être plus disposées à participer aux enquêtes locales, où elles ont peut-être moins peur de dévoiler une orientation sexuelle ou une identité de genre minoritaire que dans le cadre d’une enquête gouvernementale. Nous avons cherché à savoir si la proportion des personnes 2S/GBTQ+ qui dévoileraient leur orientation sexuelle pour une enquête gouvernementale a changé entre les cycles d’enquête de 2012 et de 2019, ainsi que la proportion de personnes participantes trans, non binaires et bispirituelles qui révéleraient leur identité de genre, et les facteurs démographiques associés aux deux populations.

**Méthodes:**

Nous avons analysé les données des cycles de 2012 et de 2019 de Sexe au présent, un sondage en ligne transversal pancanadien sur la santé et le bien-être des personnes 2S/GBTQ+ mené par le Centre de recherche communautaire. Nous avons calculé en fonction d’un éventail de variables démographiques la fréquence et les ratios de prévalence de la probabilité de dévoiler son orientation sexuelle et son identité de genre dans le cadre d’une enquête de Statistique Canada.

**Résultats:**

Nous avons constaté qu’en 2019, 86%, IC de 95% [85,4, 86,7], des personnes participantes auraient révélé leur orientation sexuelle, ce qui représente une hausse importante par rapport à 2012 (69,5%, IC de 95% [68,5, 70,4], Δ=16.6%, IC de 95% [15,4, 17,8]). Cependant, les hommes s’identifiant comme étant bisexuels, hétérosexuels ou hétéroflexibles; qui étaient en couple avec une femme; ou qui n’étaient pas « sortis du placard» étaient moins disposés à révéler leur orientation sexuelle. Selon nos constatations, 85% des hommes trans et des personnes non binaires et bispirituelles auraient révélé leur identité de genre, un résultat qui était plus probable chez les personnes vivant avec le VIH ou âgées de 19 à 29 ans.

**Conclusion:**

Ces constats indiquent que les fichiers du gouvernement pourraient significativement mal classifier et sous-estimer la taille de la population 2S/GBTQ+. La méfiance persistante envers les institutions gouvernementales dans cette communauté pourrait exacerber la sous-déclaration et la non-divulgation, d’où la nécessité de faire de la recherche sur les méthodes susceptibles de renforcer la confiance et d’améliorer l’exactitude des estimations de cette population. Les chercheurs et les chercheuses qui utilisent les fichiers gouvernementaux existants devraient songer à utiliser des méthodes statistiques pour tenir compte des erreurs de classification possibles.

## Introduction

Government-conducted population surveys are essential for understanding health inequities among marginalized groups, including gay, bisexual, transgender, and queer men and nonbinary and Two-Spirit people (2S/GBTQ+). However, these surveys are vulnerable to several forms of information bias, including measurement bias associated with self-reported sexual orientation and gender identity (Wolff et al., 2017). Selection bias associated with different sampling strategies also impacts study findings. Probability samples of sexual minorities tend to include more married/partnered individuals and fewer individuals who are employed, lesbian/gay-identified, and report higher substance use, suicidal ideation, and sexual partners (Salway et al., 2019). United States (US) government survey samples of trans people have included more individuals who are older, married, heterosexual, and Black or Hispanic compared to non-probability samples (Bauerband et al., 2023; Lett & Everhart, 2021). While sexual orientation and gender identity questions have historically been stigmatized, nonresponse rates for sexual orientation questions have improved over time (Bates et al., 2019).

While there is little research on how trans, nonbinary, and Two-Spirit people respond to government surveys, transphobia and cisnormativity likely shape individuals’ willingness to disclose their gender (Suen et al., 2022). Many population health surveys use the two-step gender measure, which Statistics Canada implemented for the first time in the 2021 Census (Statistics Canada, 2022). This method compares participants’ gender identity and sex assigned at birth to identify trans respondents (Lombardi & Banik, 2016). The Trans PULSE Canada survey asked trans and nonbinary participants to answer sex and gender questions as if completing a Statistics Canada survey and found that 82% of participants would be correctly classified as trans or nonbinary based on their responses (The Trans PULSE Canada Team, 2022). Other studies suggest that while most trans people are willing to disclose their trans identity in government health research, they have concerns regarding question wording and intent (Bauer et al., 2017; Holzberg et al., 2017).

Understanding the prevalence of non-disclosure and misclassification of sexual and gender minority identities on government surveys is important, as these biases can affect study validity and findings (Salway et al., 2015, 2019). For instance, Salway et al. (2015) argued that misclassification errors contribute to underestimating the burden of HIV mortality among men who have sex with men. As government surveys are key authoritative sources of population health data, understanding how sexual and gender minority individuals respond to these surveys is critical to accurately interpreting and applying their findings. If particular demographic groups of 2S/GBTQ+ people are less likely to disclose their sexual and gender identities in government health surveys, these groups’ health needs will not be accurately represented in the data, and 2S/GBTQ+ health programs and policies may not meet their needs. Using data from the 2012 edition of Sex Now, a Canada-wide bilingual periodic cross-sectional survey of GBTQ+ men, nonbinary, and Two-Spirit people, Ferlatte and colleagues (2017) found that 86% of gay participants and 40% of bisexual participants were willing to disclose their sexual orientation on a Statistics Canada survey. However, public acceptance of sexual minorities in Canada has increased considerably since 2012 (Flores, 2021), which may increase 2S/GBTQ+ people’s willingness to disclose their sexual orientation in a government survey. To investigate this possibility, we analysed data from the 2019 edition of Sex Now and compared it with data from the 2012 edition. We also considered additional predictors and added an analysis of disclosure of gender identity. Our research questions were (1) What proportion of participants indicated they would disclose their sexual orientation and gender identity on a Statistics Canada survey, and does this vary by demographic variables?; and (2) Is this proportion significantly different from Sex Now 2012?

## Methods

### Data

Sex Now is a Canada-wide bilingual periodic cross-sectional survey conducted by the Community-Based Research Centre (CBRC) to promote the health and well-being of 2S/GBTQ+ people. We analysed data from Sex Now 2012 and 2019. In both years, data were collected anonymously using an online survey promoted on dating/sex-seeking websites and apps, social media, word-of-mouth, and community partner organizations (Salway et al., 2016, 2021). In 2012, men who have sex with men aged 18 and over living in Canada were eligible to participate (Ferlatte et al., 2017). In 2019, participants had to (i) identify as a man (including trans man), nonbinary, or Two-Spirit; (ii) identify as gay, bisexual, queer, or another non-heterosexual identity or report having had sex with a man in the last five years; and (iii) be 15 or older and living in Canada (Salway et al., 2021). Trans women were ineligible. Participants provided consent to participate before completing the survey, which was available in English and French.

### Dependent variables

Our primary dependent variable was derived from a question that asked participants how likely they would be to reveal their sexual orientation in a Statistics Canada survey. In 2012, this question was phrased as: “Would you reveal your sexual orientation if asked in a Statistics Canada survey?” In 2019, this question was phrased as “How likely or unlikely would you be to reveal each of the following, if asked in a Statistics Canada survey (e.g., Census, Canadian Community Health Survey)?” and had separate questions for “sexual orientation” and “gender identity (i.e., cisgender, transgender, non-binary)”. The response options were “Very likely,” “Likely,” “Unlikely,” and “Very unlikely” in 2019, and similar in 2012 (“Somewhat unlikely” and “Totally unlikely” were used instead). We recoded both variables into a binary “Likely” and “Unlikely” variable for analyses.

### Independent variables

Our independent variables of interest were survey year, province of residence, age (at the time of the survey, at the time of coming out, and at first sex with a man), gender identity, trans status, sexual identity, relationship status, rurality, income, education, HIV status, and ethnicity. As question wording and response options in the 2012 and 2019 surveys were not identical, we recoded some variables to create compatible categories (see Supplemental Table 1). We further collapsed some categories for the gender identity disclosure analysis due to the smaller sample size.

### Analyses

We merged the 2012 and 2019 datasets into a single dataset and conducted all analyses using SAS 9.4. We computed frequencies of disclosing sexual orientation if asked in a Statistics Canada survey by each of our independent variables and conducted a Pearson’s chi-squared test (*α* = 0.05) to assess the change between 2012 and 2019 of the percent of participants who said they would disclose their sexual orientation. Next, only for trans, nonbinary, and Two-Spirit participants, we computed frequencies of disclosing gender identity if asked in a Statistics Canada survey by each of our independent variables. We calculated prevalence ratios and their 95% confidence intervals for each of our independent variables using PROC GENMOD’s log-binomial regression (Spiegelman & Hertzmark, 2005). Specifically, we conducted binary contrasts, where we compared each category to the weighted mean of all other categories (Johfre & Freese, 2021).

## Results

### Prevalence of revealing sexual orientation in 2012

In 2012, 49.8%, 95% CI [48.7, 50.9] (*n* = 4173), of participants said they were very likely to reveal their sexual orientation if asked in a Statistics Canada survey; 19.7% [18.8, 20.5] (*n* = 1649) said they were likely to do so, 11.3% [10.7, 12.0] (*n* = 950) were somewhat unlikely to disclose, and 19.1% [18.4, 20.1] (*n* = 1610) were totally unlikely to disclose. Likelihood of disclosure differed significantly by sexual orientation, with 85.9% [85.0, 86.4] of gay participants indicating they would disclose, compared with only 38.8% [36.9, 40.6] of bisexual participants and 34.3% [27.3, 41.3] of heterosexual participants. Participants who were currently in a relationship with a woman were much less likely to indicate they would disclose their sexual orientation than participants who were single, in a relationship with a man, or had another relationship status (see Table 1). Further, only 37.0% [35.3, 38.7] of participants who said they were not out about their sexuality to other guys said they would disclose. We also observed differences in willingness to disclose by the age at which participants came out and the age at which they first had sex with a man, with participants who came out or had sex younger being more likely to disclose. Prevalence of disclosing sexual orientation also varied by participants’ province of residence, gender identity, trans status, age, rurality, income, education, HIV status, and ethnicity. The largest differences were based on HIV status and age, with younger participants and participants living with HIV being more likely to disclose.

**Table 1:** Self-reported likelihood of disclosing sexual orientation in Statistics Canada survey by demographic variables

Sex Now 2012				Sex Now 2019				Change from 2012 to 2019		
Variable	Total* n*	Likely^a^ % (95% CI)	PR of likely (95% CI)	Variable	Total *n*	Likely^a^ % (95% CI)	PR of likely (95% CI)	Δ likely (95% CI)	χ^2^	*p*
Overall	8382	69.5 (68.5, 70.4)		Overall	10,415	86.0 (85.4, 86.7)		16.6 (15.4, 17.8)	761.31	** <.0001**
Province/Territories				Province/Territories						
Alberta	1064	66.8 (64.0, 69.7)	0.96 (0.90, 1.02)	Alberta	1573	83.5 (81.6, 85.3)	0.99 (0.96, 1.02)	16.7 (13.3, 20.0)	98.59	** <.0001**
British Columbia	1804	75.0 (73.0, 77.0)	**1.09 (1.03, 1.14)**	British Columbia	1833	87.5 (86.0, 89.0)	**1.04 (1.01, 1.07)**	12.5 (10.0, 15.0)	93.56	** <.0001**
Manitoba	342	62.3 (57.1, 67.4)	**0.89 (0.81, 0.97)**	Manitoba	319	85.6 (81.7, 89.4)	1.01 (0.96, 1.07)	23.3 (16.9, 29.7)	46.03	** <.0001**
New Brunswick	105	72.4 (63.8, 80.9)	1.04 (0.92, 1.19)	New Brunswick	188	81.4 (75.8, 86.9)	0.96 (0.89, 1.03)	9.0 (−1.2, 19.2)	3.20	0.0737
Newfoundland & Labrador	85	71.8 (62.2, 81.3)	1.04 (0.90, 1.19)	Newfoundland & Labrador	175	88 (83.2, 92.8)	1.05 (0.99, 1.11)	16.2 (5.5, 27.0)	10.54	**0.0012**
Nova Scotia	223	76.7 (71.1, 82.2)	**1.11 (1.02, 1.21)**	Nova Scotia	463	87.3 (84.2, 90.3)	1.04 (0.99, 1.08)	10.6 (4.3, 16.9)	12.41	**0.0004**
Ontario	3366	66.5 (64.9, 68.0)	0.95 (0.91, 1.00)	Ontario	3162	83.7 (82.5, 85.0)	0.99 (0.96, 1.02)	17.3 (15.2, 19.3)	257.88	** <.0001**
Prince Edward Island	33	72.7 (57.5, 87.9)	1.05 (0.85, 1.30)	Prince Edward Island	50	82 (71.4, 92.6)	0.97 (0.85, 1.10)	9.3 (−9.3, 27.8)	1.01	0.3158
Quebec	1048	74.3 (71.7, 77.0)	**1.08 (1.02, 1.14)**	Quebec	2330	90.6 (89.4, 91.8)	**1.08 (1.05, 1.11)**	16.3 (13.4, 19.2)	154.80	** <.0001**
Saskatchewan	289	62.6 (57.1, 68.2)	**0.89 (0.81, 0.99)**	Saskatchewan	274	81.4 (76.8, 86.0)	0.96 (0.90, 1.02)	18.8 (11.5, 26.0)	24.42	** <.0001**
Territories	23	65.2 (45.8, 84.7)	0.93 (0.69, 1.26)	Territories	48	79.2 (67.7, 90.7)	0.93 (0.80, 1.08)	14.0 (−36.6, 8.7)	1.60	0.2061
Gender				Gender						
Man	8299	69.4 (68.4, 70.4)	0.96 (0.84, 1.10)	Man	9992	85.7 (85.0, 86.4)	**0.92 (0.89, 0.94)**	16.3 (15.1, 17.5)	709.58	** <.0001**
Other	83	72.3 (62.7, 81.9)	1.04 (0.91, 1.19)	Other	419	93.5 (91.2, 95.9)	**1.09 (1.07, 1.12)**	21.3 (11.4, 31.2)	34.94	** <.0001**
Trans status				Trans status						
Trans	98	77.7 (69.3, 85.8)	**1.12 (1.00, 1.25)**	Trans	914	89.4 (87.4, 91.4)	**1.04 (1.02, 1.07)**	11.8 (3.3, 20.3)	11.95	**0.0002**
Not trans	8284	69.4 (68.4, 70.4)	**0.89 (0.80, 0.996)**	Not trans	9350	85.8 (85.1, 86.5)	**0.96 (0.94, 0.98)**	16.4 (15.2, 17.6)	689.96	** <.0001**
				Two-Spirit (among Indigenous participants)						
				Indigenous Two-Spirit	199	94 (90.7, 97.3)	**1.16 (1.09, 1.23)**			
				Indigenous non-Two-Spirit	320	81.3 (77.0, 85.5)	**0.86 (0.81, 0.92)**			
Sexuality				Sexuality						
Gay (homosexual)	5406	85.9 (85.0, 86.4)	**1.81 (1.67, 1.96)**	Gay (only)	6139	93 (92.3, 93.6)	**1.29 (1.23, 1.34)**	7.1 (5.9, 8.2)	154.32	** <.0001**
Straight (heterosexual)	175	34.3 (27.3, 41.3)	**0.53 (0.43, 0.66)**	Straight	144	62.5 (54.6, 70.4)	**0.76 (0.67, 0.86)**	28.2 (17.6, 38.8)	25.24	** <.0001**
Bi (bisexual)	2720	38.8 (36.9, 40.6)	**0.63 (0.57, 0.69)**	Bisexual	2402	66.3 (64.4, 68.2)	**0.82 (0.78, 0.86)**	27.6 (24.9, 30.2)	388.26	** <.0001**
Other	81	80.3 (71.6, 88.9)	**1.65 (1.45, 1.88)**	Other	1696	91.3 (89.9, 92.6)	**1.25 (1.20, 1.31)**	11.0 (2.3, 19.8)	11.22	** <.0001**
				Asexual	178	84.3 (78.9, 89.6)	0.98 (0.92, 1.04)			
				Pansexual	786	86 (83.6, 88.4)	1.00 (0.97, 1.03)			
				Queer	1530	94.2 (93.1, 95.4)	**1.11 (1.10, 1.13)**			
				Heteroflexible	259	62.9 (57.1, 68.8)	**0.72 (0.66, 0.80)**			
Relationship status				Relationship status						
Single	4222	72.5 (71.2, 73.9)	**1.15 (1.10, 1.19)**	Single	4590	86.5 (85.5, 87.5)	**1.13 (1.10, 1.16)**	13.9 (12.3, 15.6)	265.37	** <.0001**
Partnered with a man	2215	91.3 (90.2, 92.5)	**1.56 (1.50, 1.62)**	Partnered with a man	4212	93.9 (93.2, 94.6)	**1.26 (1.23, 1.29)**	2.5 (1.2, 3.9)	14.41	**0.0001**
Partnered with a woman	1798	34.4 (32.2, 36.6)	**0.42 (0.40, 0.45)**	Partnered with a woman	1116	53 (50.1, 56.0)	**0.56 (0.56, 0.62)**	18.7 (15.0, 22.3)	98.9038	** <.0001**
Other	147	80.3 (73.8, 86.7)	**1.31 (1.21, 1.43)**	Other (in relationship with nonbinary person or 1+ person)	497	89.7 (87.1, 92.4)	**1.19 (1.15, 1.23)**	9.5 (2.5, 16.4)	9.3444	**0.0022**
Age group (in years)				Age group (in years)						
18 or younger	132	79.5 (72.7, 86.4)	**1.15 (1.05, 1.26)**	18 or younger	361	82.5 (78.6, 86.5)	0.98 (0.93, 1.03)	3.0 (−4.91, 10.9)	0.5841	0.4447
19–29	1734	78.1 (76.2, 80.1)	**1.12 (1.08, 1.17)**	19–29	3274	89.6 (88.6, 90.7)	**1.09 (1.06, 1.11)**	11.5 (9.3, 13.7)	121.34	** <.0001**
30–59	5466	67.8 (66.6, 69.0)	**0.93 (0.89, 0.97)**	30–59	5890	85.3 (84.4, 86.3)	1.02 (0.99, 1.04)	17.6 (16.0, 19.1)	491.43	** <.0001**
60 or older	1050	62.5 (59.5, 65.4)	**0.83 (0.79, 0.88)**	60 or older	890	79 (81.7, 23.7)	**0.92 (0.88, 0.96)**	16.5 (12.6, 20.5)	62.61	** <.0001**
Rurality				Rurality						
Rural	1245	60.8 (58.1, 63.5)	**0.86 (0.82, 0.90)**	Rural area (< 1000 people)	424	79 (75.1, 82.9)	**0.90 (0.85, 0.94)**	17.1 (15.9, 18.4)	46.35	** <.0001**
Non-rural	7137	71.0 (69.9, 72.0)	**1.17 (1.11, 1.22)**	Non-rural (1000+ people)	8491	88.1 (87.4, 88.8)	**1.11 (1.06, 1.17)**	18.2 (13.5, 22.9)	719.29	** <.0001**
Income (annual, CAD)				Income (annual, CAD)						
Under $10,000	739	74.3 (71.1, 77.4)	**1.08 (1.03, 1.13)**	< $10,000	786	89.2 (87.0, 91.4)	1.01 (0.98, 1.03)	14.9 (11.1, 18.7)	57.18	** <.0001**
$10,000—$19,999	779	73.2 (70.1, 76.3)	**1.06 (1.02, 1.11)**	$10,000–$19,999	1084	90.7 (89.0, 92.4)	**1.02 (1.00, 1.05)**	17.5 (14.0, 21.1)	100.21	** <.0001**
$20,000—$29,999	874	75.3 (72.4, 78.1)	**1.10 (1.05, 1.14)**	$20,000–$29,999	926	90 (88.0, 91.9)	1.01 (0.99, 1.04)	14.7 (11.2, 18.1)	68.06	** <.0001**
$30,000—$49,999	1847	68.3 (66.2, 70.4)	0.98 (0.95, 1.02)	$30,000–$49,999	1743	90.1 (88.7, 91.5)	1.02 (1.00, 1.04)	21.9 (19.3, 24.4)	257.25	** <.0001**
$50,000—$59,999	968	71.5 (68.6, 74.3)	1.04 (0.99, 1.08)	$50,000–$59,999	756	88.2 (85.9, 90.5)	0.99 (0.97, 1.02)	16.7 (13.1, 20.4)	71.27	** <.0001**
$60,000—$69,999	730	68.5 (65.1, 71.9)	0.99 (0.94, 1.04)	$60,000–$69,999	613	86.9 (84.3, 89.6)	0.98 (0.95, 1.01)	18.5 (14.2, 22.8)	63.93	** <.0001**
$70,000—$79,999	608	66.9 (63.2, 70.7)	0.96 (0.91, 1.02)	$70,000–$79,999	527	88.8 (86.1, 91.5)	1.00 (0.97, 1.03)	21.9 (17.3, 26.5)	76.42	** <.0001**
$80,000—$89,999	484	65.7 (61.5, 69.9)	0.94 (0.88, 1.01)	$80,000–$89,999	431	91.2 (88.5, 93.9)	1.03 (1.00, 1.06)	25.5 (20.5, 30.5)	85.44	** <.0001**
$90,000—$99,999	345	67.0 (62.0, 71.9)	0.96 (0.89, 1.04)	$90,000–$99,999	306	87.3 (83.5, 91.0)	0.98 (0.94, 1.02)	20.3 (14.1, 26.5)	37.16	** <.0001**
$100,000+	1007	63.2 (60.2, 66.1)	**0.90 (0.86, 0.95)**	$100,00	976	86 (83.8, 88.1)	**0.96 (0.94, 0.99)**	22.8 (19.1, 26.5)	135.27	** <.0001**
Education				Education						
Some high school	348	59.8 (54.6, 64.9)	**0.90 (0.82, 0.99)**	Did not finish high school	361	82 (78.0, 86.0)	**0.94 (0.90, 0.99)**	22.2 (15.7, 28.7)	42.58	** <.0001**
High school	1131	61.7 (58.9, 64.5)	0.95 (0.89, 1.01)	High school, or equivalent	1733	85.5 (83.9, 87.2)	1.00 (0.97, 1.04)	23.8 (20.5, 27.1)	213.26	** <.0001**
Any post-secondary	6903	71.2 (70.1, 72.3)	**1.17 (1.11, 1.23)**	Any post-secondary	6774	88.5 (87.8, 89.3)	**1.06 (1.03, 1.09)**	20.2 (16.0, 18.6)	634.37	** <.0001**
HIV status (self-reported)				HIV status (self-reported)						
HIV negative or unknown	7715	67.8 (66.7, 68.8)	**0.76 (0.74, 0.79)**	Never been diagnosed with HIV	6673	88.3 (87.6, 89.1)	**0.94 (0.92, 0.96)**	20.6 (19.3, 21.9)	862.16	** <.0001**
HIV positive	667	89.1 (86.7, 91.4)	**1.31 (1.27, 1.35)**	Living with HIV	609	93.6 (91.7, 95.5)	**1.06 (1.04, 1.08)**	4.5 (1.5, 7.6)	8.20	**0.0042**
Ethnicity				Ethnicity						
Indigenous	168	73.2 (66.5, 79.9)	1.05 (0.95, 1.16)	Indigenous	558	86.4 (83.5, 89.2)	1.00 (0.96, 1.04)	13.2 (5.9, 20.4)	16.11	** <.0001**
ACB	76	67.1 (56.6, 77.7)	0.94 (0.80, 1.10)	ACB	361	86.4 (82.9, 90.0)	1.00 (0.96, 1.04)	19.3 (8.2, 30.5)	16.66	** <.0001**
Asian	286	69.2 (63.9, 74.6)	0.98 (0.89, 1.07)	Asian	739	85.1 (82.5, 87.7)	0.98 (0.95, 1.01)	15.9 (10.0, 21.8)	33.38	** <.0001**
Other POC	542	74.0 (70.3, 77.7)	1.06 (0.99, 1.14)	Other POC	600	88.3 (85.8, 90.9)	1.03 (0.99, 1.06)	14.4 (9.9, 18.9)	38.92	** <.0001**
White only	7310	69.1 (68.0, 70.1)	0.98 (0.92, 1.03)	White only	8017	86.1 (85.3, 86.8)	0.99 (0.98, 1.01)	17.0 (15.7, 18.3)	642.92	** <.0001**
Age when first had sex with man				Age when first had sex with man						
< 16	3091	72.7 (71.1, 74.2)	**1.08 (1.05, 1.11)**	< 16	2454	86.9 (85.6, 88.3)	**1.02 (1.00, 1.05)**	14.3 (12.2, 16.3)	167.43	** <.0001**
16–18	1896	75.4 (73.4, 77.3)	**1.13 (1.10, 1.17)**	16–18	3028	90.2 (89.2, 91.3)	**1.07 (1.05, 1.09)**	14.9 (12.7, 17.0)	196.50	** <.0001**
19–22	1462	73.1 (70.8, 75.4)	**1.09 (1.05, 1.13)**	19–22	2318	89.3 (88.0, 90.5)	**1.06 (1.04, 1.08)**	16.1 (13.5, 18.7)	165.62	** <.0001**
23+	1897	55.6 (53,4, 57.9)	**0.75 (0.72, 0.79)**	23+	1717	78.9 (77.0, 80.8)	**0.91 (0.88, 0.93)**	23.3 (20.4, 26.3)	220.29	** <.0001**
				Never had sex with a man	366	81.4 (77.4, 85.4)	**0.94, 0.90, 0.99)**			
Age when came out				Age when came out						
< 16	489	93.9 (91.7, 96.0)	**1.32 (1.28, 1.35)**	< 16	1512	93.5 (92.2, 94.7)	**1.17 (1.14, 1.12)**	−0.4 (−2.9, 2.1)	0.10	0.7467
16–18	1281	90 (88.4, 91.7)	**1.25 (1.22, 1.28)**	16–18	2327	94.6 (93.7, 95.5)	**1.19 (1.17, 1.21)**	4.6 (2.7, 6.5)	27.05	** <.0001**
19–22	1342	90.6 (89.1, 92.2)	**1.26 (1.23, 1.29)**	19–22	2289	93.2 (92.2, 94.2)	**1.16 (1.14, 1.18)**	2.6 (0.7, 4.4)	7.82	**0.0052**
23+	2179	84.9 (83.4, 86.4)	**1.16 (1.14, 1.19)**	23+	2480	91.9 (90.8, 93.0)	**1.14 (1.12, 1.16)**	7.0 (5.2, 8.8)	56.27	** <.0001**
Not out	3091	37 (35.3, 38.7)	**0.41 (0.39, 0.43)**	Not out	1585	50.7 (48.3, 53.2)	**0.54 (0.52, 0.57)**	13.7 (10.7, 16.7)	81.09	** <.0001**
				Degree of outness						
				1 (Not at all open/out)	998	34.6 (31.6, 37.5)	**0.42 (0.39, 0.46)**			
				2	748	61.6 (58.1, 65.1)	**0.86 (0.81, 0.92)**			
				3	1118	85 (82.9, 87.1)	**1.29 (1.24, 1.34)**			
				4	1653	92.1 (90.8, 93.4)	**1.43 (1.34, 1.47)**			
				5 (Open/out to all or most people)	5881	96.4 (95.9, 96.8)	**1.51 (1.47, 1.55)**			

^a^“Likely” includes participants who indicated they were “Very likely” or “Likely” to disclose their sexual orientation. *HIV* refers to human immunodeficiency virus. *ACB* means African, Caribbean, and/or Black. *POC* means person of colour. *PR* means prevalence ratio. *CI* means confidence interval

Bolded text emphasizes results that are statistically significant at *p*<0.05

### Prevalence of revealing sexual orientation in 2019

In 2019, the majority (68.4%, 95% CI [67.5, 69.3], *n* = 7121) of participants said they were very likely to disclose their sexual orientation if asked in a Statistics Canada survey; 17.7% [16.9, 18.4] (*n* = 1841) said they were likely to do so, 7.1% [6.6, 7.6] (*n* = 744) said they were unlikely to do so, and 6.8% [6.3, 7.3] (*n* = 709) said they were very unlikely to do so. Overall, willingness to disclose increased significantly by 16.6% ([15.4, 17.8], *p* < 0.0001) from 2012. Bivariate analysis revealed that while the prevalence of disclosing sexual orientation increased across all demographic variables, some differences persisted, especially in terms of sexual orientation and relationship status. Only 62.5% [54.6, 70.4] of straight, 62.9% [57.1, 68.8] of heteroflexible participants, and 66.3% [64.4%, 68.2%] of bisexual participants said that they would disclose their sexual orientation, compared with 93.0% [92.3, 93.6] of participants who identified solely as gay. Similarly, just over half of participants in a relationship with a woman said they would disclose their sexual orientation—much fewer than participants who were single or in a relationship with a man, with a nonbinary person, or with more than one person. Further, only 34.6% [31.6, 37.5] of participants who were not at all open/out about their sexuality said they would disclose their sexual orientation, compared with 96.4% [95.9, 96.8] of those who were open/out to all or most people. Likelihood of disclosing sexual orientation also varied slightly by province of residence, gender identity, trans status, being Two-Spirit, age, rurality, income, educational attainment, age at first sex with a man, age of coming out, and HIV status. Overall, these findings indicate that bisexual, straight, and heteroflexible participants, participants in a relationship with a woman, and participants who were not out were still less likely to reveal their sexual orientation in 2019. However, a comparison of prevalence ratios from 2012 and 2019 indicates that the gap has decreased.

We conducted a post-hoc logistic regression to assess whether a change in participant demographics impacted our findings, and found that the increased willingness to disclose in 2019 persisted after controlling for demographic variables (see Supplementary Information).

### Prevalence of revealing gender identity in 2019

In 2019, the majority (62.9%, 95% CI [60.1, 65.7],* n* = 723) of participants who identified as trans, nonbinary, and/or Two-Spirit said they were very likely to disclose their gender identity if asked in a Statistics Canada survey. Additionally, 22.1% [19.7, 24.5] (*n* = 254) said they were likely to reveal. Only 9.8% [8.1, 11.5] of participants (*n* = 113) said they were unlikely to reveal, and a further 5.2% [3.9, 6.5] (*n* = 60) said they were very unlikely to reveal. Prevalence ratios indicated that participants living with HIV and participants aged 19–29 were slightly more likely to reveal their gender identity (see Table 2). Within the group of trans, nonbinary, and/or Two-Spirit participants analysed here, there were no statistically significant differences by gender identity, being Two-Spirit, or trans status.

**Table 2:** Likelihood of disclosing gender identity in Statistics Canada survey by demographic variables, for trans, nonbinary, and/or two-spirit participants in Sex Now 2019

	Total *n*	Likely^a^ (%)	95% CI of %	PR of likely (95% CI)
Province/region				
Atlantic	34	85.3	(73.4, 97.2)	1.01 (0.87, 1.18)
British Columbia	234	85.0	(80.5, 89.6)	1.01 (0.93, 1.10)
Ontario	275	85.1	(80.9, 89.3)	1.01 (0.93, 1.10)
Prairies	340	84.4	(80.5, 88.2)	0.99 (0.93, 1.08)
Quebec	152	88.2	(83.0, 93.3)	1.05 (0.67, 1.15)
Territories	14	78.6	(57.0, 100.0)	0.92 (0.70, 1.21)
Age group (in years)				
15–18	89	79.8	(71.4, 88.1)	0.96 (0.86, 1.07)
19–29	580	88.1	(85.5, 90.7)	**1.10 (1.03, 1.16)**
30–49	364	83.5	(79.7, 87.3)	1.02 (0.96, 1.09)
50+	117	77.8	(70.2, 85.3)	0.93 (0.84, 1.03)
Trans				
No	200	86.5	(81.8, 91.2)	1.02 (0.96, 1.09)
Yes	922	84.6	(82.3, 86.9)	0.98 (0.92, 1.04)
Gender				
Man	736	83.8	(81.2, 86.5)	0.96 (0.91, 1.11)
Nonbinary	340	86.8	(83.2, 90.4)	1.01 (0.95, 1.08)
Other	73	87.7	(80.1, 95.2)	1.03 (0.94, 1.13)
Two-Spirit (among Indigenous participants)				
Indigenous non-Two-Spirit	72	79.2	(69.7, 88.6)	0.90 (0.79, 1.02)
Indigenous Two-Spirit	200	88.0	(83.5, 92.5)	1.11 (0.98, 1.23)
Relationship status (by partner gender)				
No	503	85.5	(82.4, 88.6)	1.01 (0.94, 1.07)
Yes, with a man	325	84.3	(80.3, 88.3)	1.00 (0.94, 1.06)
Yes, with a woman	144	80.6	(74.1, 87.0)	0.94 (0.86, 1.02)
Yes, with a nonbinary person or more than one person	178	88.2	(83.5, 92.9)	1.06 (0.99, 1.13)
Rurality				
Non-rural	931	86.3	(84.0, 88.5)	1.09 (0.95, 1.24)
Rural	58	79.3	(68.9, 89.8)	0.92 (0.80, 1.05)
Income (personal, CAD)				
< $10,000	198	87.9	(83.3, 92.4)	1.03 (0.97, 1.10)
$10,000–$19,999	240	86.3	(81.9, 90.6)	1.01 (0.94, 1.07)
$20,000–$50,000	148	81.8	(75.5, 88.0)	0.94 (0.86, 1.02)
$50,000+	113	87.6	(81.5, 93.7)	1.03 (0.95, 1.11)
Education				
Did not finish high school	78	85.9	(78.2, 93.6)	1.00 (0.91, 1.10)
High school or equivalent	325	85.5	(81.7, 89.4)	1.00 (0.93, 1.06)
Any post-secondary education	574	85.9	(83.0, 88.7)	1.00 (0.94, 1.06)
HIV status (self-reported)				
No (never been diagnosed with HIV)	786	86.0	(83.6, 88.4)	**0.92 (0.85, 0.9981)**
Yes (living with HIV)	46	93.5	(86.3, 100)	**1.09 (1.00, 1.18)**
Ethnicity				
Indigenous	283	85.2	(69.0, 96.5)	1.01 (0.94, 1.09)
African, Caribbean, Black	29	82.8	(77.7, 95.9)	0.98 (0.82, 1.16)
Asian (including East, South, and Southeast Asian)	53	86.8	(81.0, 89.3)	1.04 (0.91, 1.17)
Other POC (Latin American and Arab/West Asian)	49	81.6	(70.8, 92.5)	0.96 (0.83, 1.11)
White only	708	85.3	(82.7, 87.9)	1.01 (0.95, 1.09)

^a^Likely includes participants who indicated they were “Very likely” or “Likely” to disclose their gender identity. *HIV* means human immunodeficiency virus. *POC* means person of colour. *PR* means prevalence ratio. *CI* means confidence interval

Bolded text emphasizes results that are statistically significant at *p*<0.05

## Discussion

We found that the proportion of 2S/GBTQ+ participants who said they would disclose their sexual orientation in a Statistics Canada survey increased significantly between 2012 and 2019, from 70 to 86%. However, 14% of participants were still unlikely to reveal their sexual orientation in 2019. Specifically, bisexual, straight, and heteroflexible participants, and participants who were in a relationship with a woman, and/or not out were much less likely to reveal their sexual orientation. Our findings suggest that estimates based on government survey data that do not account for non-disclosure underestimate the 2S/GBTQ+ population size (Sorge et al., 2023), which has important implications for prevalence estimates and population health responses.

Interpreting our finding that only 63% of straight participants would reveal their sexual orientation requires considering how participants conceptualized sexual orientation. While definitions vary, sexual orientation is a multi-dimensional construct that encompasses identity, attraction, and behaviours. These aspects of sexuality may change for individuals across their lifespan and are not always aligned in normative ways (van Anders, 2015). As the Sex Now surveys did not define sexual orientation, we cannot be certain how participants interpreted this question. However, straight-identified Sex Now participants were likely indicating a reluctance to disclose same-gender attractions or behaviours, rather than a heterosexual identity. Qualitative research could explore how straight-identified men who have sex with men interpret the term “sexual orientation.” Individuals with branched sexualities^1^ whose sexual identity, behaviour, and attractions do not align in normative ways—such as straight-identified men who have sex with men—may experience unique health risk factors and stressors (Lefevor et al., 2022; Mendelsohn et al., 2022). Other participants who were less likely to disclose their sexual orientation, such as individuals who are bisexual or not out, may also have unique health profiles. Reliable data that capture multiple facets of sexual orientation are needed to better understand the health profiles of our diverse population.

Our findings suggest that single-item sexual identity measures misclassify a significant proportion of straight-identified men who have sex with men as heterosexual. While Statistics Canada recognizes that sexual orientation is not synonymous with sexual attraction or behaviour, the agency’s standard adopted in 2023 only measures “how a person describes their sexuality,” such as “heterosexual, lesbian, gay, bisexual or pansexual” (Statistics Canada, 2023). However, the 2015-2016 edition of their Canadian Community Health Survey included questions about both sexual behaviours and identity. Analyzing this data, Silva and Fetner (2022) found that 20% of men who had ever engaged in sexual behaviours with other men identified as heterosexual. Similarly, Dharma and Bauer (2017) estimated that 16% of individuals who had ever engaged in same-sex sexual behaviour would be classified as heterosexual by Statistics Canada’s single-item sexual identity question. If we interpret our finding that 37% of straight-identified men who have sex with men would not disclose their sexual orientation to Statistics Canada to mean that they would not disclose their same-sex sexual behaviour, likely even fewer of these men would be categorized as sexual minorities than previously estimated.

Our findings underscore the need for additional research on sexual minority health in Canada using more expansive measures of sexual orientation. Researchers should carefully choose sexual orientation measures that align with their research questions. Asking about both sexual identity and behaviours may help better identify straight-identified men who have sex with men, though many may still not disclose their sexual behaviours. To promote trust and transparency, researchers should inform participants of how and why sexual orientation data will be used in analysis and highlight safety and confidentiality protections (Puckett et al., 2020; Suen et al., 2022). Meaningful and reciprocal partnerships with trusted community leaders and organizations may be necessary to ameliorate distrust based on historic harms. When analysing existing government data, our findings about the prevalence of non-disclosure and misclassification of sexual and gender minority individuals can inform statistical adjustments for these biases, such as those developed by Dharma et al. (2025).

In terms of gender, we found that 15% of trans men, nonbinary people, and Two-Spirit Sex Now survey participants were unlikely to reveal their gender identity in a Statistics Canada survey, suggesting that estimates of this population will likely be an undercount. Notably, the 2021 Census introduced a new measure of sex and gender, which asked individuals their sex at birth (male or female) and their gender (male, female, or other, where other includes a write-in text box) (Statistics Canada, 2022). As we only asked participants their general willingness to reveal their gender identity in a Statistics Canada survey, we cannot be sure how participants would have responded to the specific 2021 Census questions (see The Trans PULSE Canada Team, 2022 for this). Some individuals who were willing to disclose their gender identity may have been unwilling to report their sex at birth (Puckett et al., 2020) and might therefore be misclassified as cisgender. Future research should explore disclosure of gender modality, expression, and other sex/gender-related constructs. Further, anti-trans social attitudes and political attacks have risen significantly since 2019, and future research should explore how this shapes willingness to disclose gender identity in government surveys.

Some research indicates that trans participants prefer to be asked their gender identity before being asked their sex at birth (Lombardi & Banik, 2016). However, the limited available evidence has not found that the order of these questions impacts nonresponse bias (DeChants et al., 2021; Saperstein & Westbrook, 2021). The validity of any measure of gender identity will likely be impacted by participants’ understanding of the question’s purpose and planned usage (Holzberg et al., 2017; Suen et al., 2022). Researchers seeking to represent trans people accurately and respectfully in their surveys should refer to Lowik et al.’s guidelines *Gender and Sex in Methods and Measurement Research Equity Toolkit* (2022).

Another important concern is how Indigenous Two-Spirit people are represented in government health surveys. Per Pruden and Salway (2020), Two-Spirit is a term that serves as a “community organizing tool for Indigenous Peoples of Turtle Island who embody diverse sexualities, gender identities, roles, and/or expressions” that “challenges Western terms of gender and sexual orientation.” As such, it cannot be treated as simply a gender or sexuality (Pruden & Salway, 2020). Neither the sexual orientation nor gender questions currently used by Statistics Canada ask individuals whether they are Two-Spirit. Statistics Canada (2022) indicates that anyone who reported their gender as Two-Spirit in the “other” response option of the 2021 Census is included in the category “non-binary persons.” The sexual orientation standard adopted in 2023 classifies individuals who write in their sexual orientation as Two-Spirit as “sexual orientation, not elsewhere classified” and includes them in the broader LGB+ category. Based on these standards, Statistics Canada survey data does not allow researchers to conduct analyses specifically on Two-Spirit people. While concerns about the identifiability of small populations are important, collapsing Two-Spirit people into larger LGBTQ categories perpetuates the colonial erasure of Two-Spirit people and obscures their distinct experiences and needs (Lowik et al., 2022). Researchers designing surveys should consider Pruden and Salway’s (2020) recommendations to ask about being Two-Spirit after a question about race and ethnicity, and only ask the Two-Spirit question to Indigenous participants.

### Limitations

Our research has several limitations. First, Sex Now has historically been promoted as about “sex between guys.” While non-heterosexual trans men and nonbinary people were eligible to participate regardless of recent sex with men, the survey eligibility and recruitment strategies limit representation from the full diversity of trans and nonbinary communities. Similarly, we caution extrapolation of Sex Now data to the entire 2S/LGBTQ+ community given the explicit exclusion of lesbian, bisexual, queer, and trans women. Second, we measured intent to disclose on a future survey rather than past behaviour. As such, we do not know how individuals actually responded to Statistics Canada surveys, which may be impacted by factors such as question wording (e.g., asking sex at birth and gender identity, as noted above). Third, our non-probability sample was largely recruited from social media or dating platforms and may underrepresent individuals who are not connected to online 2S/GBTQ+ communities, though less so than samples recruited in person. Previous research has found that non-probability samples tend to overrepresent some people, such as gay-identified individuals, and underrepresent others, such as married and partnered individuals (Salway et al., 2019). As gay identification was associated with a higher likelihood of disclosure and being in a relationship with a woman with a lower likelihood, the true proportion of 2S/GBTQ+ people who would disclose their sexual orientation on a Statistics Canada survey may be even lower than we have reported. Fourth, we did not run multivariable models to assess whether demographic variables were independently associated with willingness to disclose when controlling for other variables. Finally, our question on willingness to disclose specified collection on a Statistics Canada survey, which may limit generalizability to other federal government bodies, other levels of government, or other countries.

## Conclusion

The proportion of 2S/GBTQ+ survey participants who would reveal their sexual orientation in a Statistics Canada survey increased significantly from 2012 to 2019, from 70 to 86%. However, fewer participants who identified as bisexual, straight, or heteroflexible, as well as participants who were in a relationship with a woman or who were not out about their sexuality would reveal their sexual orientation. Additionally, 85% of trans men, nonbinary, and Two-Spirit Sex Now survey participants said they would reveal their gender identity in a Statistics Canada survey, with few significant differences by demographic variables. These findings indicate that Statistics Canada datasets likely underestimate the 2S/GBTQ+ population by approximately 15%. Population health research must attend to information bias introduced by non-disclosure of stigmatized information. This requires both technical adjustments in analyses and proactive trust- and relationship-building.

## **Contributions to knowledge**

### What does this study add to existing knowledge?

- The proportion of 2S/GBTQ+ survey participants who would disclose their sexual orientation in a federal government survey increased significantly from 2012 to 2019, from 70 to 86%. Participants who identified as bisexual, straight, or heteroflexible; were in relationship with a woman; or were not out about their sexuality were less willing to disclose their sexual orientation.
- 85% of trans men, nonbinary, and Two-Spirit participants would reveal their gender identity in a federal government survey.

### What are the key implications for public health interventions, practice, or policy?

- Government datasets that use self-reported sexual identity and gender identity will undercount 2S/GBTQ+ individuals, impacting population size estimates. Willingness to disclose sexual orientation varies by demographic factors that have important implications for measuring, understanding, and intervening in health inequities.

## Supplementary Information

Below is the link to the electronic supplementary material.

ESM 1 Supplementary Material 1 (DOCX 19.5 KB)

## Data Availability

Partial Sex Now data is publicly available on the OurStats dashboard (https://ourstats.ca/our-dashboard). Data access requests can be made through the protocol outlined at: https://www.cbrc.net/cbrc_data_access_process

## Footnotes

1 We use the terms “branched” and “coincident” rather than “discordant” and “concordant” to avoid using language that implies a value judgement about normative sexualities, as per van Anders (2015).

## Code availability

SAS code for analyses is available upon request.
