# Supplementary material for: Willingness to disclose sexual orientation and gender identity on federal government surveys: A community-based research study with gay, bisexual, transgender, and queer men and nonbinary and Two-Spirit people in Canada
Source: Can J Public Health. 2025 Oct 17;117(3):564–75. doi: 10.17269/s41997-025-01127-0 (PMC13337983; doi:10.17269/s41997-025-01127-0)
Supplement: Supplementary file 1 — Supplementary Material 1 (DOCX 19.5 KB) [file 41997_2025_1127_MOESM1_ESM.docx]

# Supplementary Material

To test whether the increased proportion of participants in 2019 who were likely to disclose their sexual orientation was due to a change in sample demographics, we conducted a post-hoc logistic regression. The primary independent variable was survey year and the dependent variable was likelihood of disclosing sexual orientation. To select demographic variables to control for, we compared the previously identified independent variables between the 2012 and 2019 samples using a Chi-squared test to identify changes. We found significant differences between 2012 and 2019 for province, gender, trans status, sexual identity, relationship status, age, rurality, income, education, ethnicity, age at first sex with man, and age of coming out (all *p* < .0001). We did not find a significant difference in HIV status between 2012 and 2019 (*p* = .2875). We therefore controlled for all independent variables except HIV status in the logistic regression model. We found that 2019 participants had 1.98 times the odds (95% CI = [1.76, 2.22], *p* <.0001) of disclosing sexual orientation compared to 2012 participants, indicating our results are not primarily due to differing sample demographics.

**Supplementary Table 1**

*Recoded Variables*

| ***Variable*** | ***Sexual Orientation Analysis*** | ***2012 Survey Response Options*** | ***2019 Survey Response Options*** | ***Gender Identity Analysis*** |
| --- | --- | --- | --- | --- |
| **Gender identity** | Man | Man; Transman | Man | Man |
|  | Other | Transwoman; Other | Prefer to self-describe, recoded to nonbinary | Nonbinary |
|  |  |  | Prefer to self-describe, recoded to other | Other |
| **Trans status** | Yes | Transman; Transwoman; Other | “Do you have trans experience? (i.e., your gender is different than the sex you were assigned at birth)” = Yes | Yes |
|  | No | Man | “Do you have trans experience? (i.e., your gender is different than the sex you were assigned at birth)” = No | No |
| **Sexual identity** | Gay | Gay (homosexual) | Gay (only selection) | n/a |
|  | Straight | Straight (heterosexual) | Straight |  |
|  | Bi | Bi (bisexual) | Bi (bisexual) (regardless of other selections, unless Gay selected) |  |
|  | Other | Other | Asexual; Pansexual; Queer; Heteroflexible; Other |  |
| **Relationship status** | Single | Single; Separated, divorced from a man; Separated, divorced from a woman | Are you currently in a relationship? (committed or non-committed) - No | Are you currently in a relationship? (committed or non-committed) - No |
|  | Partnered with a man | Married to a man; Partnered with a man but not married | Are you currently in a relationship? (committed or non-committed) – Yes, with a man | Are you currently in a relationship? (committed or non-committed) – Yes, with a man |
|  | Partnered with a woman | Married to a woman; Partnered with a woman but not married | Are you currently in a relationship? (committed or non-committed) – Yes, with a woman | Are you currently in a relationship? (committed or non-committed) – Yes, with a woman |
|  | Other | Other | Are you currently in a relationship? (committed or non-committed) – Yes, with a nonbinary person; Yes, with more than 1 person (polyamorous) | Are you currently in a relationship? (committed or non-committed) – Yes, with a nonbinary person; Yes, with more than 1 person (polyamorous) |
| **Rurality** | Rural | What best describes your environment – Rural; Remote | What best describes the environment you live in? – Rural area (<1,000 people) | What best describes the environment you live in? – Rural area (<1,000 people) |
|  | Non-rural | What best describes your environment – Urban, Suburban, Other | What best describes the environment you live in? – Large urban centre (100,000+ people); Medium city/town (30,000-99,999 people); Small city/town (1,000-29,999 people) | What best describes the environment you live in? – Large urban centre (100,000+ people); Medium city/town (30,000-99,999 people); Small city/town (1,000-29,999 people) |
| **HIV Status** | HIV negative/unknown | What was your latest HIV test result? – HIV-Negative; I’ve never had an HIV test (or a test result) | Have you EVER been diagnosed with HIV? – No (I have never been diagnosed with HIV) | Have you EVER been diagnosed with HIV? – No (I have never been diagnosed with HIV) |
|  | HIV positive | What was your latest HIV test result? – HIV-Positive | Have you EVER been diagnosed with HIV? – Yes (I am living with HIV) | Have you EVER been diagnosed with HIV? – Yes (I am living with HIV) |
| **Ethnicity** | Indigenous | How do you describe yourself to other guys? – Aboriginal | Which of these do you identify with? (check all that apply) – Indigenous | Which of these do you identify with? (check all that apply) – Indigenous |
|  | ACB | How do you describe yourself to other guys? – African; Caribbean | Which of these do you identify with? (check all that apply) – African; Black; Caribbean (Indigenous not checked) | Which of these do you identify with? (check all that apply) – African; Black; Caribbean (Indigenous not checked) |
|  | Asian | How do you describe yourself to other guys? – Asian, South Asian | Which of these do you identify with? (check all that apply) – East Asian (e.g. Chinese, Japanese, Korean); South Asian (e.g. East Indian, Pakistani, Sri Lankan); Southeast Asian (e.g. Filipino, Vietnamese, Thai); (Indigenous, African, Black, Caribbean not checked) | Which of these do you identify with? (check all that apply) – East Asian (e.g. Chinese, Japanese, Korean); South Asian (e.g. East Indian, Pakistani, Sri Lankan); Southeast Asian (e.g. Filipino, Vietnamese, Thai); (Indigenous, African, Black, Caribbean not checked) |
|  | Other POC | How do you describe yourself to other guys? – Latino/Hispanic; Middle Eastern; Pacific Islander; Mixed; Other | Which of these do you identify with? (check all that apply) – Arab, West Asian (e.g. Iranian, Afghan); Latin American, Hispanic; Other (Indigenous, African, Black, Caribbean, East Asian, South Asian, Southeast Asian not checked) | Which of these do you identify with? (check all that apply) – Arab, West Asian (e.g. Iranian, Afghan); Latin American, Hispanic; Other (Indigenous, African, Black, Caribbean, East Asian, South Asian, Southeast Asian not checked) |
|  | White only | White-Caucasian | White (no other categories checked) | White (no other categories checked) |
